# Supplementary material for: Non-decameric NLRP3 reveals a TGN/MTOC-distal pathway of inflammasome activation
Source: Nat Commun. 2026 May 30;17:4866. doi: 10.1038/s41467-026-72627-x (PMC13226650; doi:10.1038/s41467-026-72627-x)
Supplement: Supplementary file 1 — Supplementary Information [file 41467_2026_72627_MOESM1_ESM.pdf]

Supplemental material for

Non-decameric NLRP3 reveals a TGN/MTOC-distal pathway of inflammasome activation

María Mateo-Tórtola *et al*.

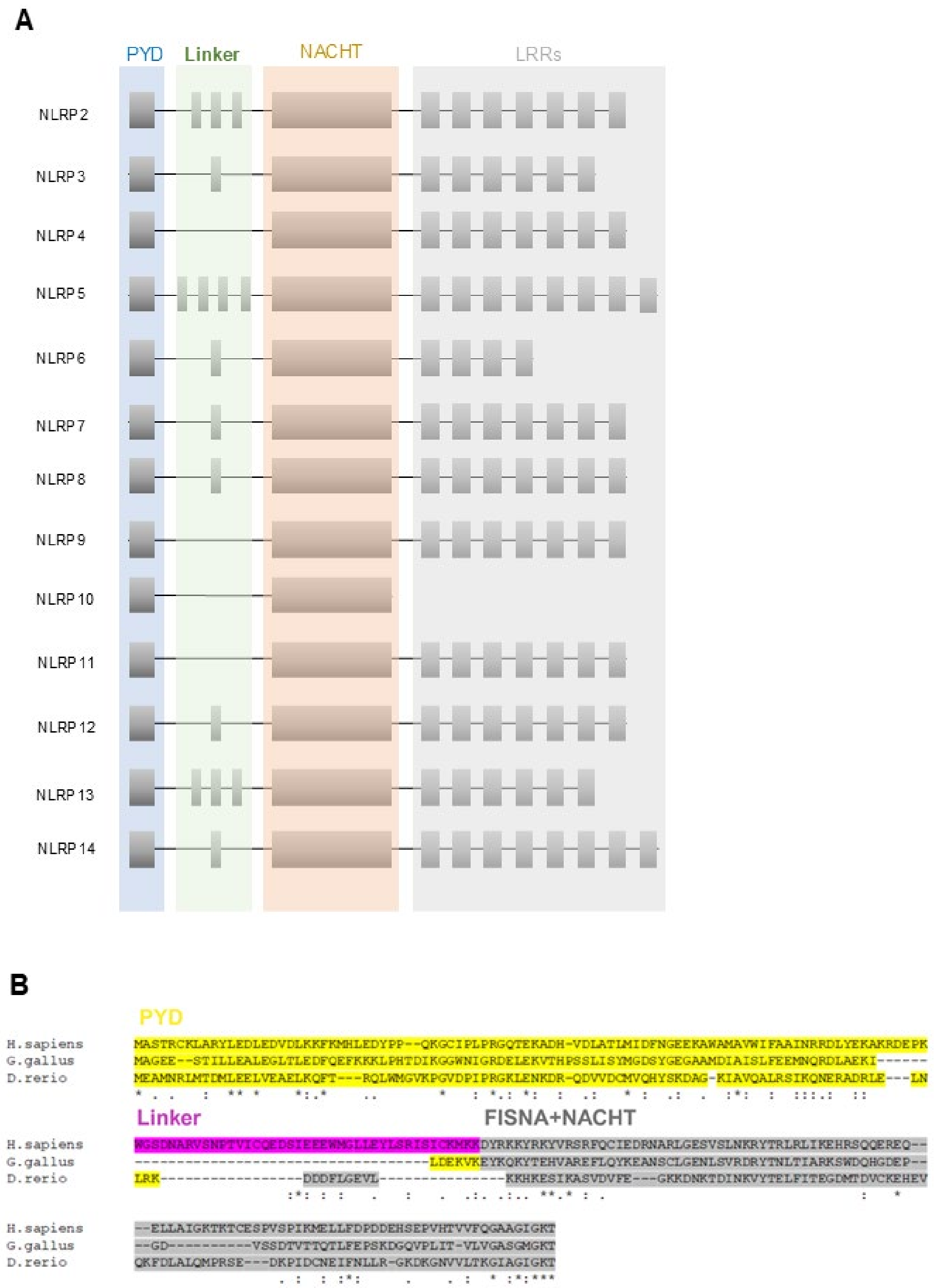

Supplementary Figure 1. Alignment of NLRPs exons. A) Alignment of human NLRPs exons with each grey box representing an exon. B) Partial alignment of human (*H. sapiens*), chicken (*Gallus gallus*) and zebrafish (*Danio rerio*) NLRP3s (Uniprot IDs: Q96P20, A0A8V0ZLM1 and A0A8M6YY09, respectively). Created in BioRender. Weber, A. (2026) https://BioRender.com/b5x4fy3

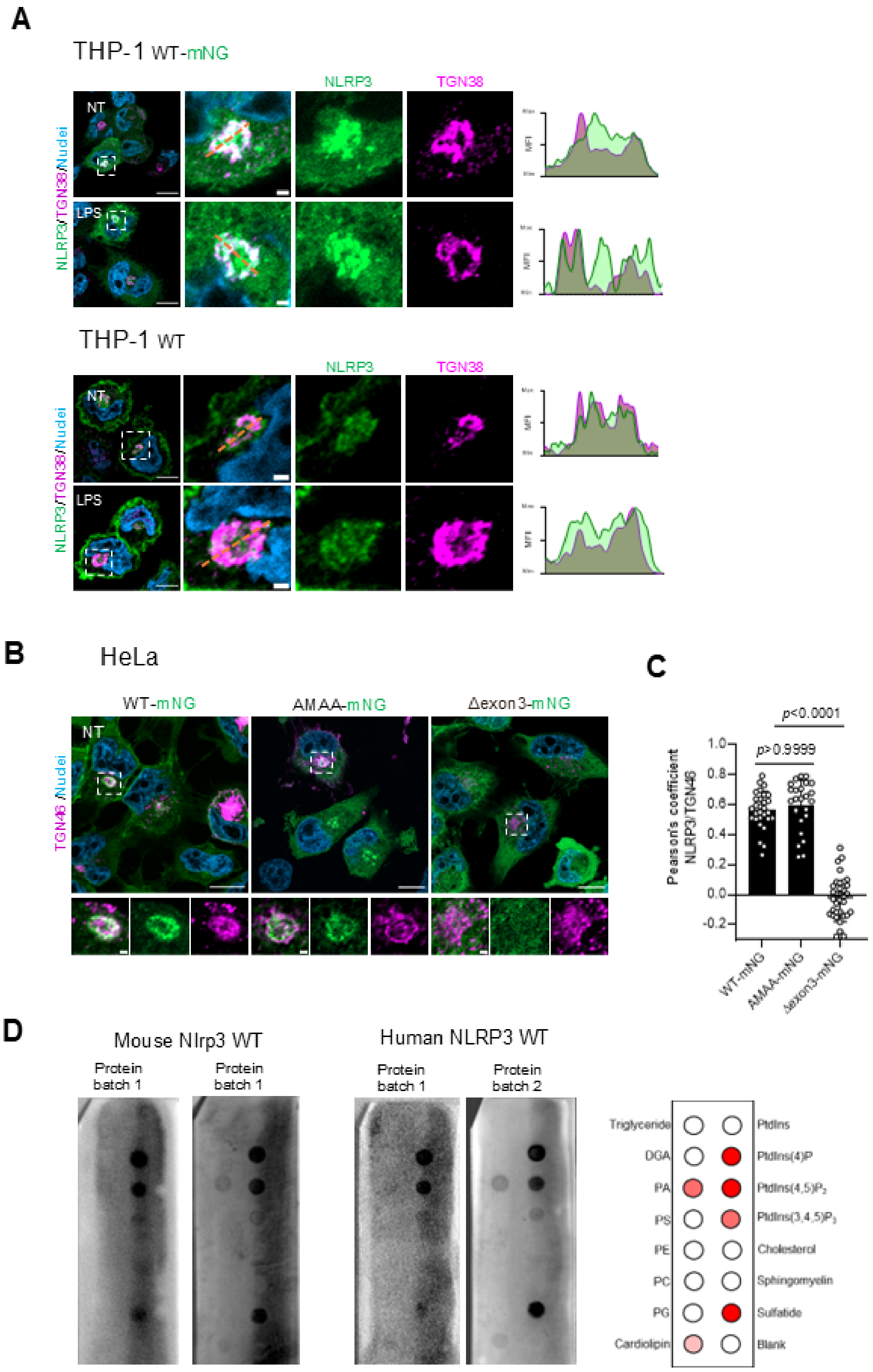

Supplementary Figure 2. Localization of endogenous NLRP3 and several NLRP3 constructs in the TGN membranes. A) Representative immunofluorescence micrographs of THP-1 NLRP3 *KO* cells reconstituted with NLRP3-mNG and THP-1 WT primed or not (non-treated, NT) with LPS at 10 ng/mL for 4 h. Endogenous NLRP3 from THP-1 WT was stained with the specific NLRP3 antibody Cryo-2 (green). The TGN for both cell lines was stained with a specific TGN38 antibody (magenta) and nuclei with Hoechst 33342 (blue). Scale bar 10 μm, 1 μm for close-ups. Hashed boxes denote close-up regions.

Representative line graphs depict changes in NLRP3 and TGN normalized MFI. N=3-4 independent experiments. B) Representative confocal microscopy images of HeLa cells stably expressing human NLRP3 WT, NLRP3 with the KMKK motif mutated to AMAA or NLRP3(Δexon3) all C-t tagged with mNG (green), and co-transfected with the TGN marker, TGN46-mCherry (magenta). Nuclei were stained with Hoechst 33342 (blue). Scale bar 10 μm, 1 μm for close-ups. N=2 independent experiments. C) Co-localization analysis of WT, AMAA or Δexon3 NLRP3 and TGN. Values are represented by Pearson´s correlation coefficient with Costes automatic thresholding. Each bar represents the mean ± SD with each data point representing one cell from 2 independent experiments (around 20 cells per independent experiment). Two-sided Kruskal-Wallis test with Dunn´s multiple comparisons test. D) In vitro lipid strip assay of purified mouse and human NLRP3. On the right, the arrangement of different lipids on the membrane is shown. Lipids that are bound are highlighted in varying shades of red, reflecting the strength of the binding signal. N=2 independent experiments.

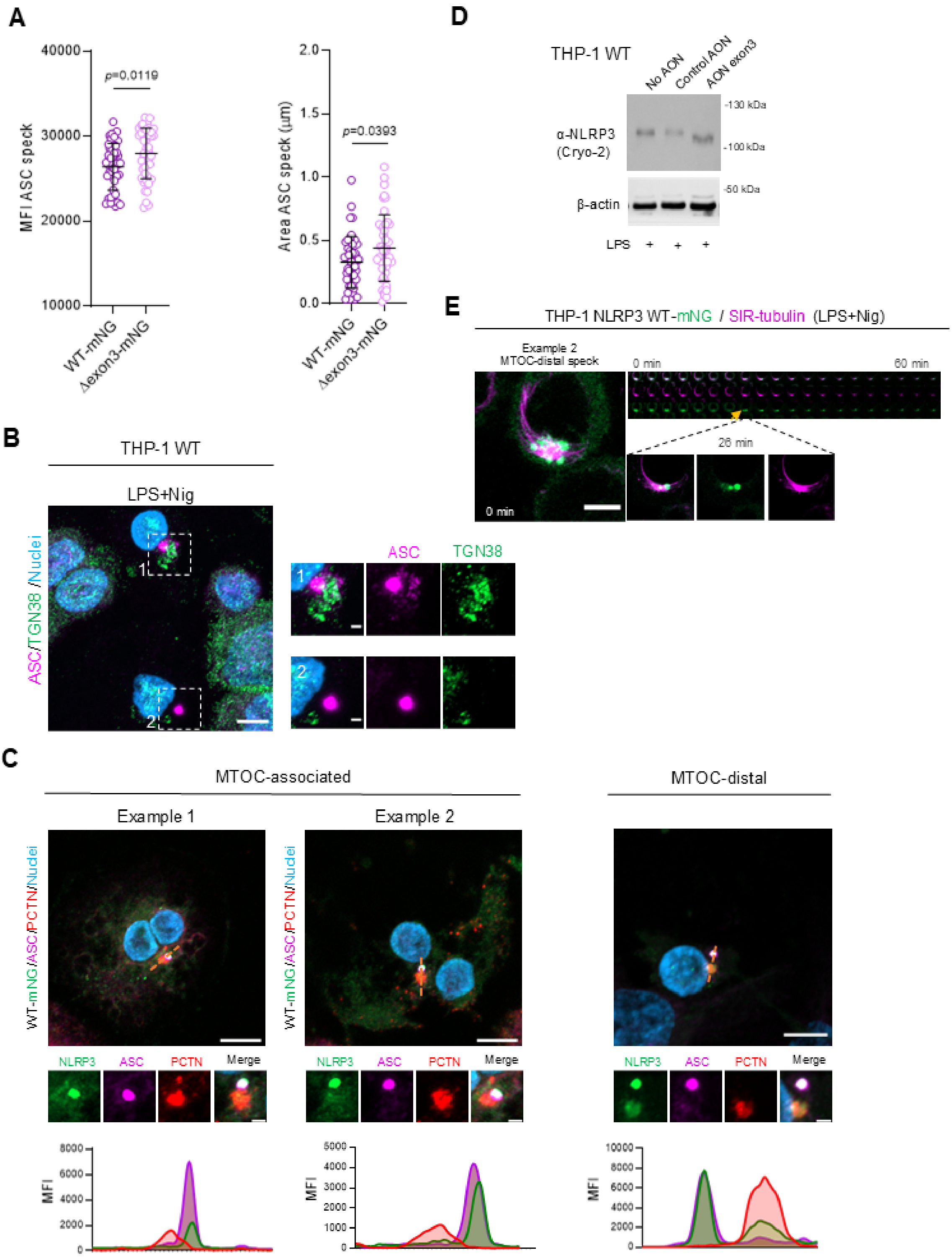

Supplementary Figure 3. ASC forms TGN- and MTOC proximal and distal specks in response to nigericin. A) Fluorescence intensity (left) and area (right) of the TGN-independent NLRP3-ASC platforms of the indicated LPS-primed and nigericin-stimulated THP-1 cell lines. Data are represented as mean ± SD and each data point represents one

speck from 3 independent experiments Two-sided Mann–Whitney U test. B) Representative immunofluorescence images of LPS-primed and nigericin-stimulated THP-1 cells with endogenous ASC stained using a specific ASC antibody (magenta) and TGN with TGN38 antibody (green). Nuclei were stained using Hoechst 33342 (blue). Scale bar 5 μm, zoom in 1 μm. N=1 independent experiment. C) Representative immunofluorescence images of LPS-primed and nigericin-stimulated NLRP3 WT-mNG THP-1 cells (green) with endogenous ASC stained using a specific ASC antibody (magenta) and MTOC with Pericentrin antibody (red). Nuclei were stained using Hoechst 33342 (blue). Scale bar 5 μm, zoom in 1 μm. N=3 independent experiments. NLRP3, ASC and pericentrin representative line graphs depicting changes in MFI are shown. D) Immunoblot of PMA-differentiated and LPS-primed THP-1 cells treated with control or NLRP3 exon 3–targeting morpholino, showing the NLRP3 band shift, revealing a lower-molecular weight NLRP3 species consistent with exon 3 skipping (N=2 independent experiments). E) Representative live cell imaging of the MTOC-dependent NLRP3 speck formation from LPS-primed and nigericin-stimulated THP-1 NLRP3 WT-mNG cells. Microtubules were stained with the specific probe SIR-tubulin (magenta). Sequential images acquired every 3 min. Scale bar 5 μm. N=3 independent experiments.

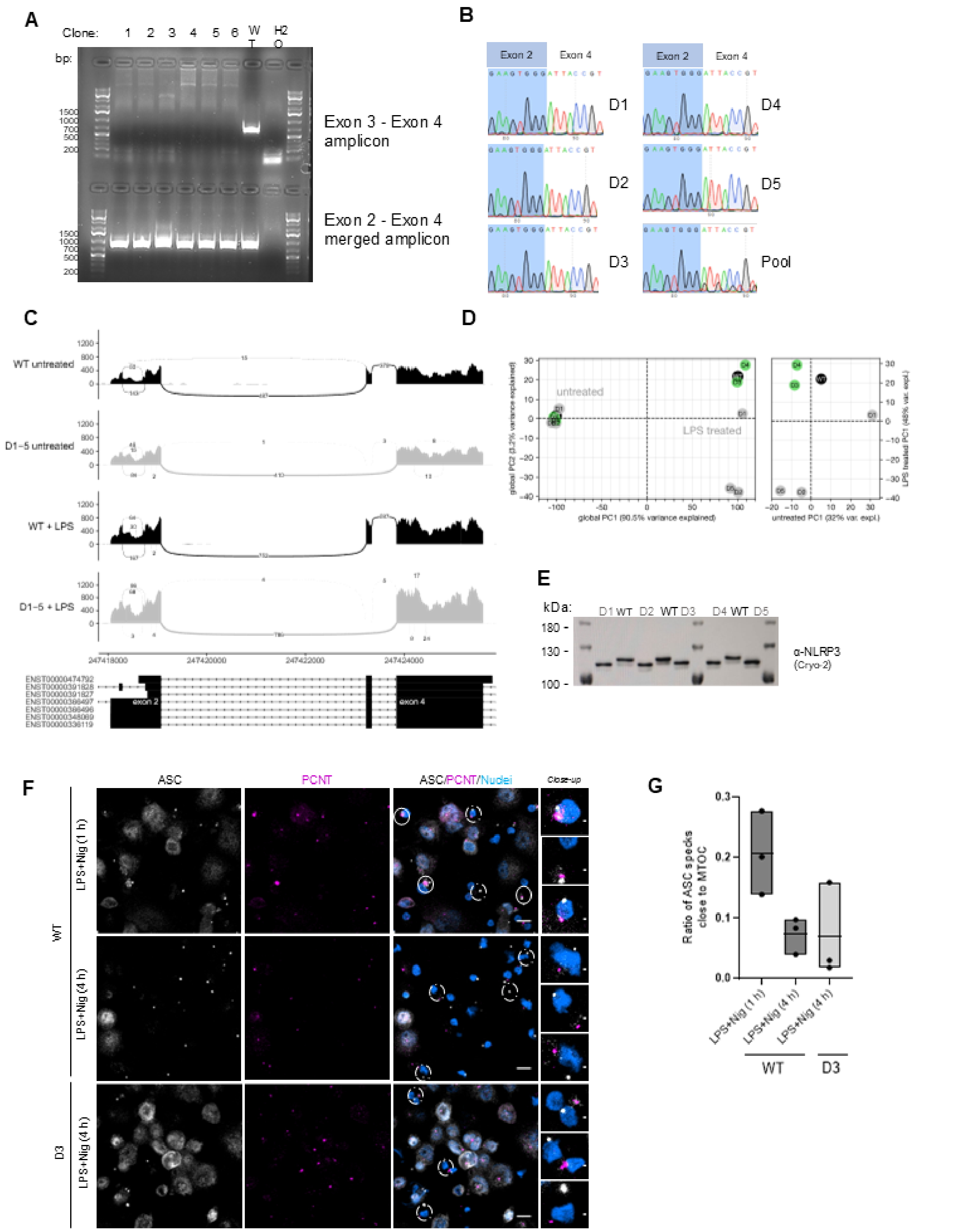

Supplementary Figure 4. Endogenous CRISPR-Cas9-engineered NLRP3 Δexon3 THP-1 clones: genomic, transcriptomic, protein and imaging validation. A) Genomic PCR analysis of WT, CRISPR-Cas9 Δexon3 edited pool, and single-cell clones (original clones 1–6). Two primer pairs were used: exon3–exon4 and exon2–exon4. Expected amplicon sizes: 660 bp (exon3–exon4) and 870 bp (exon2–exon4 merged). Original clone 3 showed a

smeared PCR product and was excluded; the remaining five clones were renamed D1–D5. B) Sanger sequencing (Microsynth AG) of the 870 bp exon2–exon4 amplicon from clones D1–D5, showing the exon2–exon4 junction. C) RNA-seq sashimi plot of the NLRP3 exon 2–4 region in WT and Δexon3 clones before and after LPS priming. Coverage tracks across exons 2–4 are shown. Δexon3 clones D1–D5 are displayed as an aggregate (mean counts). Junction arcs indicate splice junction read counts. D) PCA of WT (black circles) and Δexon3 clones D1–D5 (grey circles; D3 and D4 in green) ± LPS priming. PCA was performed on size-factor–normalized whole-transcriptome counts after arcsinh transformation (asinh(count/64)). Libraries contained 54–66M reads/sample. Left: all samples; right: PCA performed separately for untreated and LPS-treated subsets. E) Immunoblot for NLRP3 WT and Δexon3 clones. F) Representative confocal images used for automated MTOC-association analysis in the indicated cell lines and time points, showing ASC and pericentrin (PCTN) staining. Dashed circles indicate MTOC-distal specks, whereas solid circles indicate MTOC-associated specks. Scale bars: 10 µm (overview images) and 1 µm (zoomed-in close-ups). G) Quantification of ASC speck proximity to pericentrin (MTOC) using a custom analysis script (GitHub link provided in Methods), comparing indicated conditions/time points. At least 50 specks were counted per experiment and condition. Each data point represents one independent experiment (n = 3).

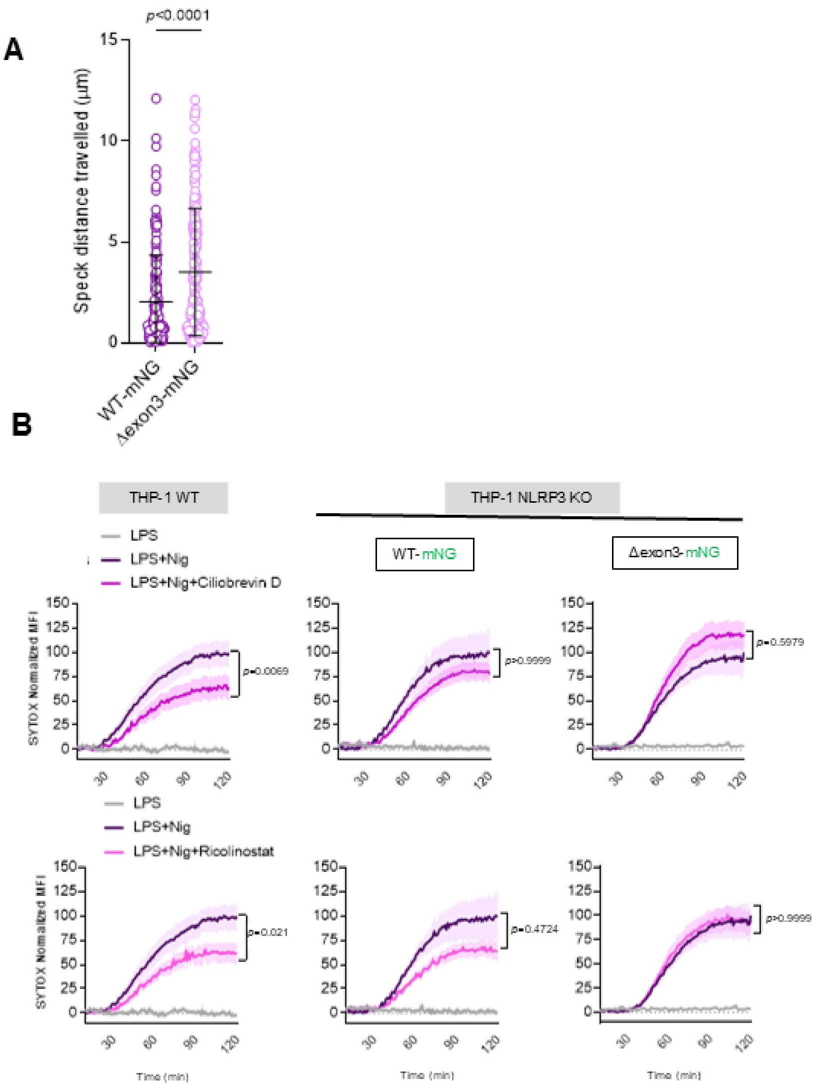

Supplementary Figure 5. NLRP3 MTOC distal specks are more motile and independent of microtubule trafficking inhibitors. A) Travelled distance (µm) by WT- or Δexon3-mNG specks in cells using Mtrack2 plugin from Fiji. Each data point represents one speck. Data are shown as mean ± SD. N=3 independent experiments (combined). Two-sided Mann-Whitney U test. **B)** Cell death in time measured by SYTOX orange uptake from the indicated THP-1 cell lines upon ciliobrevin (dynein inhibitor) and ricolinostat (HDAC6 inhibitor). Data are shown as mean ± SEM. N=3 independent experiments. Two-sided Kruskal-Wallis test with Dunn's multiple comparisons test.

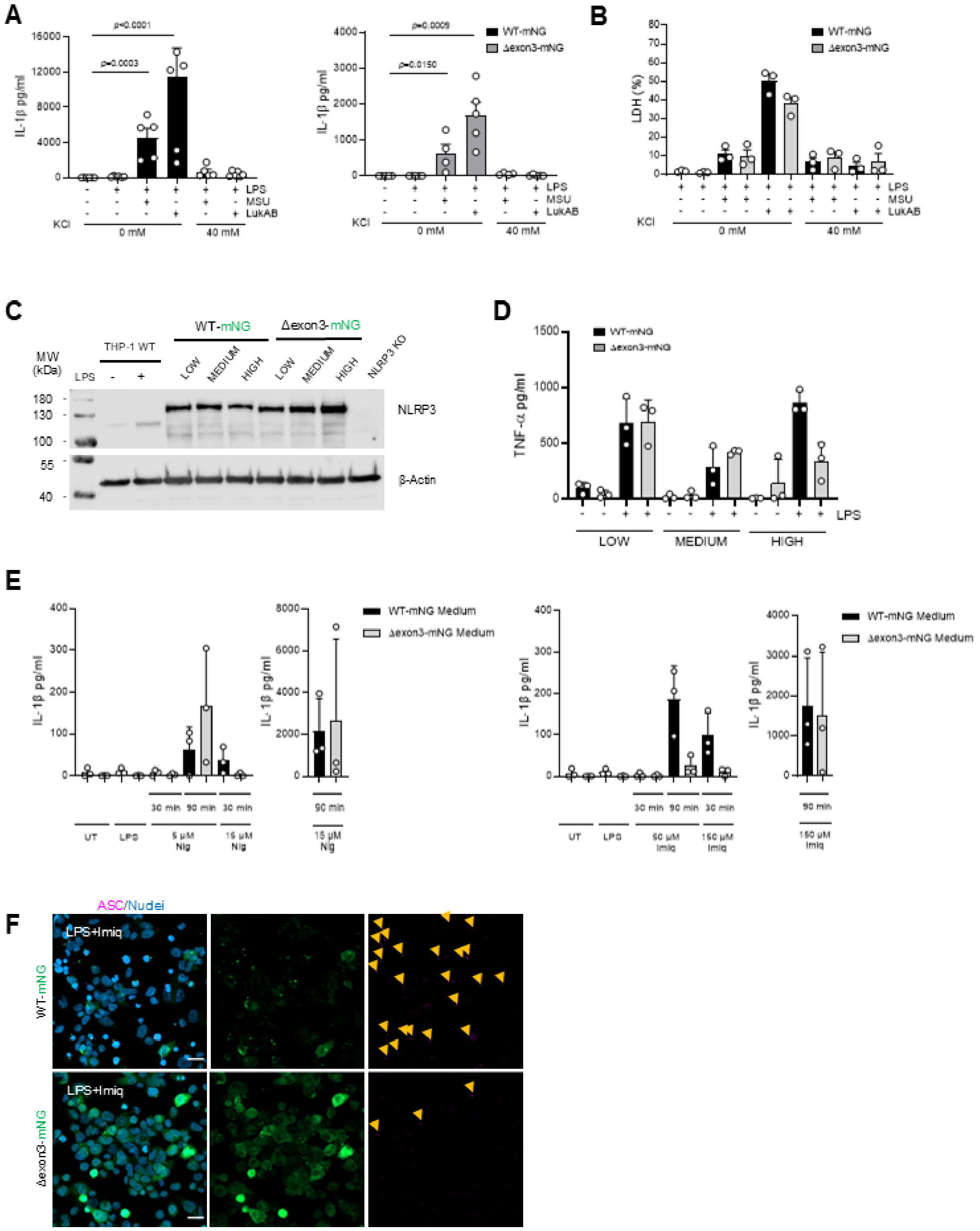

Supplementary Figure 6. K+ dependent stimuli activate both MTOC-associated and -distal NLRP3 pathways and imiquimod favors the MTOC-associated one. A, B) Reconstituted THP-1 cell lines IL-1β and LDH release after LPS-priming and 1 h stimulation with MSU crystals or LukAB in low and high K^+ conditions as indicated in the graphs. Two-sided Kruskal-Wallis test with Dunn´s multiple comparisons test. N=3-7 independent experiments (combined). C) Representatives immunoblot of sorted, LPS-primed THP-1

reconstituted cell lines classified by low, medium, or high NLRP3 expression level. β-actin served as a loading control. D) TNF-α release from cells as in B. N=3 independent experiments. E) IL-1β release from sorted THP-1 reconstituted cells with medium NLRP3 expression levels, treated as indicated in the figure. N=3 independent experiments. F) Representative immunofluorescence micrographs of ASC speck formation in reconstituted LPS-primed THP-1 cells in the presence or absence of imiquimod. Scale bar 20 μm. Arrows denote occurrence of fully formed ASC speck. N=3 independent experiments.

All data were obtained from 3 independent experiments (unless specified) and represented by mean ± SD, unless otherwise indicated

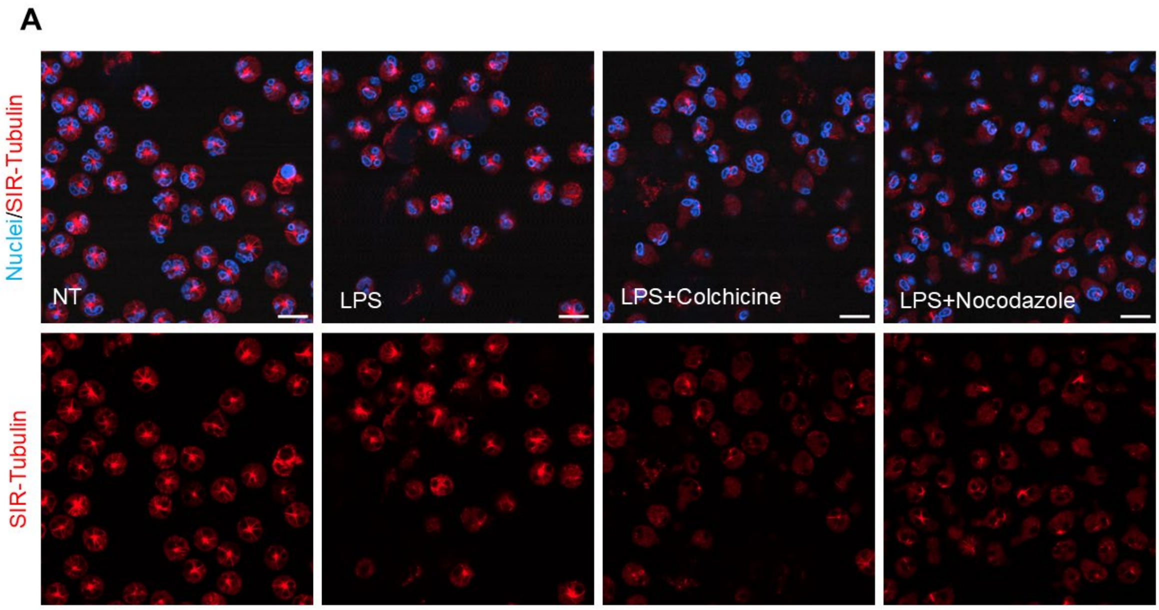

Supplementary Figure 7. Colchicine and Nocodazole disrupt the microtubule system in primary PMNs. A) Representative live cell imaging of LPS-primed human primary PMNs in the presence or absence of microtubules disruptors. Microtubules were stained with the specific probe SIR-tubulin (red). Nuclei were stained using Hoechst 33342 (blue). Scale bar 5μm.

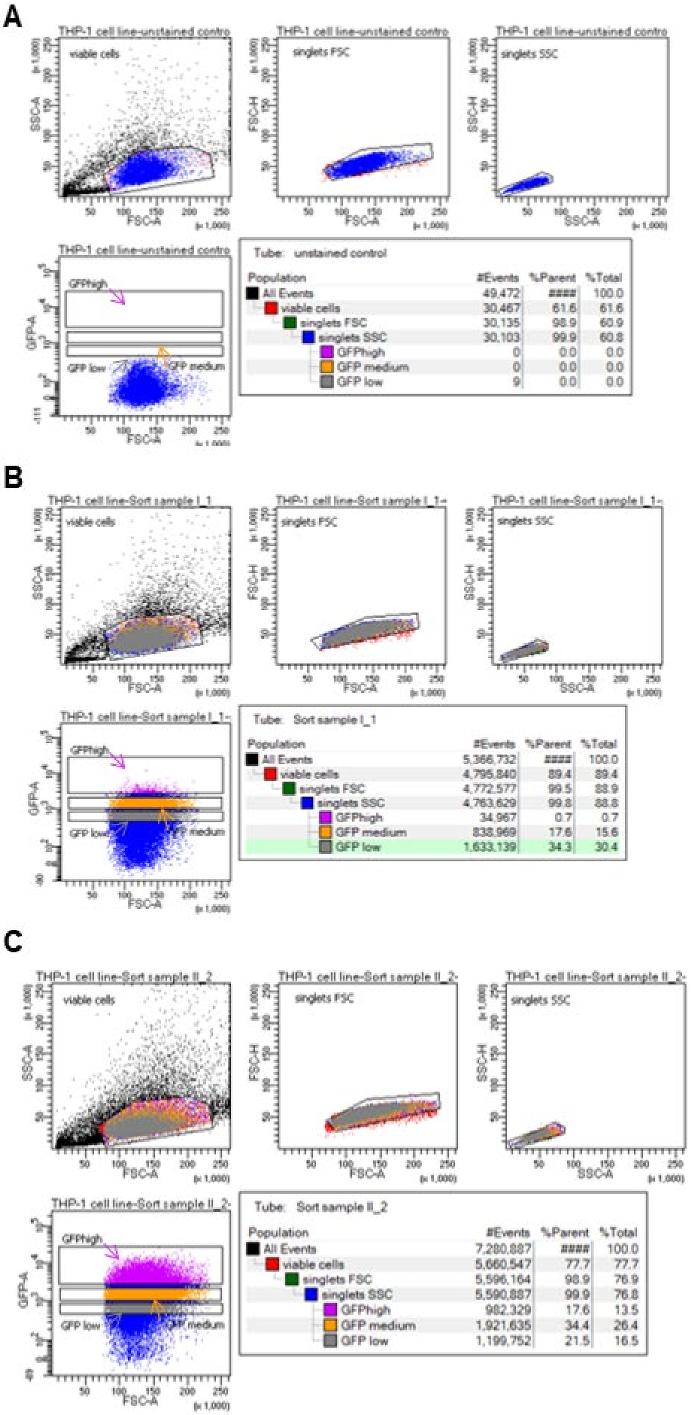

Supplementary Fig. 8. Flow cytometric gating and sorting of THP-1 NLRP3 reporter cell lines. A) Unstained THP-1 *NLRP3* KO control used to define background fluorescence and mNG gates. B) Gating and sorting strategy for THP-1 *NLRP3* KO cells expressing NLRP3 WT-mNG (sample I). C) Gating and sorting strategy for THP-1 *NLRP3* KO cells expressing Δexon3-mNG (sample II). Cells were gated based on forward and side scatter, followed by singlet discrimination, and were separated into mNG-low, mNG-medium and mNG-high fractions.

Supplementary Table 1. Main features of the MTOC-associated *vs* -distal NLRP3 pathways.

FEATURES	MTOC-associated	MTOC-distal (this study)
Subcellular localization	Microtubule-organizing center (MTOC), perinuclear clustering	Cytoplasmic, more diffusely distributed
NLRP3 oligomerization state<br>(NLRP3-mNG speck formation)	Governed by cages	Governed by smaller NLRP3 species
TGN-association<br>(TGN38 or RCAS1 staining)	Yes	No
ASC specks<br>(ASC staining)	Multiple ASC foci surrounding TGN membranes	Single ASC speck formation distal from TGN
MTOC-association<br>(tubulin or pericentrin staining)	Yes	No
Time of speck formation	Before (~30 min)	After (~60 min)
Sensitivity to microtubule disruption<br>(colchicine or nocodazole)	Yes	No
Typical in Cell Types	Macrophages, iPSC-derived microglia	Neutrophils
Additional papers providing confirming or complementary experimental evidence	Chen and Chen, Nature 2018 [1]<br>Magupalli et al; Science 2020 [2]<br>Andreeva et al; Science 2021 [3]	Hafner-Bratkovic, et al; Nat Commun 2018 [4]<br>Gluck et al; iScience 2023 [5]<br>Liu et al; Nat Commun 2023 [6]<br>Bai et al; Cell Death Discov, 2024 [7]

Supplementary Table 2. Location of NLRP3 during activation.

**ACTIVATED NLRP3**			
**Location**	**Reference**	**Method**	**Cellular system**
Mitochondria	Yang et al. 2015[8]	Confocal microscopy /Subcelullar fractionation	BMDMs/THP-1
	Elliott et al. 2018[9]	Subcelullar fractionation	J774A.1
	Subramanian et al. 2013[10]	Confocal microscopy / Subcelullar fractionation	HEK293T/BMDMs
	Kim et al. 2020[11]	Membrane fractionation	THP-1
	Misawa et al. 2013[12]	Confocal microscopy/ PLA	BMMs
	Baik et al. 2023[13]	Confocal microscopy/ Subcelullar fractionation/PLA	BMDMs
Mitochondria-associated membranes (MAMS)	Zhang et al. 2017[14]	Confocal microscopy / Subcelullar fractionation	THP-1
	Zhou et al. 2011[15]	Confocal microscopy	THP-1
Cytosol	Wang et al. 2013[16]	Confocal microscopy	Peritoneal mouse macrophages
TGN	Chen and Chen 2018 [1]	Confocal microscopy /Subcelullar fractionation	BMDMs/HEK293 cells stably expressing NLRP3-eGFP
Mitochondria-MTOC	X. Li et al. 2017 [17]	Confocal microscopy/PLA	THP-1 cells
Mitochondria-TGN	Arumugam et al. 2022[18]	Live cell imaging/ Subcellular fractionation	HeLa cells expressing fluorescent fusion proteins NLRP3-GFP
TGN-MTOC	Magupalli et al. 2020 [2]	Confocal microscopy	BMDMs
Endosomes	Zhang et al. 2023 [19]	Confocal microscopy	HeLa/THP-1 ASC KO expressing NLRP3-eGFP
Endolysosomes	Lee et al.2023[20]	Confocal microscopy	COS7 cells stably expressing NLRP3-mVenus

Supplementary Table 3. Oligonucleotide sequences used in this study for exon skipping, CRISPR-Cas9, screening PCR and qPCR.

Pimer ID	Sequence (5'-3')	Source	Target	Purpose	Reference
AWx412_F_NLRP3-Age1	GGCGCTAGCGCCACCGCCACCATGGCAAGCACCCGCTGCAAG	Biomers	NLRP3	PCR	This study
AWx413_R_NLRP3-Linker-Nhe1	GCGACCGGTCCGCTGCCAGATCCACTGCCGGACGTCCAAGAAGGCTCAAAGACGACG	Biomers	NLRP3	PCR	This study
AON exon3	CACTCCTCTTCAATGCTGTCTTCCT	Gene Tools	NLRP3 exon 3	Exon 3 skipping	This study
AON exon 5	GCTGTTCACCAATCTAGGAATTAGA	Gene Tools	NLRP3 exon 5	Exon 5 skipping	(Hoss et al., 2019)
AON control	CCTCTTACCTCAGTTACAATTTATAT	Gene Tools	Luciferase	Control exon skipping	-
TBP (housekeeper)	-	TaqMan™	TBP	qPCR	Hs00427620_m1,
NLRP3 exon 2-3	-	TaqMan™	NLRP3 exon 2-3	qPCR	Hs00918082_m1
NLRP3 exon 6-10	-	TaqMan™	NLRP3 exon 6-10	qPCR	Hs00918080_g1
GuideRNA NLRP3 exon3 skipping (exon2)	AGTGCACATAGTGTACAATT		Intron between exon 2 and exon 3 (canonical NLRP3 exons)	CRISPR-Cas9	This study
GuideRNA NLRP3 exon3 skipping (exon4)	TAGATTACCGTAAGAAGTAC		Transition intron to exon 4 (canonical NLRP3 exons)	CRISPR-Cas9	This study
NLRP3 Exon 3 Skipping CRISPR positive ctrl	GCAGACCATGTGGATCTAGC	Biomers	NLRP3 exon 2	Screening PCR	This study
NLRP3 Exon 3 Skipping CRISPR negative ctrl	GCACGTGTTTCGAATCCC	Biomers	NLRP3 exon 3	Screening PCR	This study
NLRP3 Exon 3 Skipping CRISPR reverse	CCTGTCTTCAATGCACTGG	Biomers	NLRP3 exon 4	Screening PCR	This study

Supplementary Table 4. Antibodies used in this study for immunoblotting (IB) and immunofluorescence (IF)

Antibody	Species	Isotype	Use	Company/Catalog number
anti-NLRP3 (D4D8T)	Rabbit	IgG	**IB:** 1:1000	Cell Signaling Technology, 15101
anti-NLRP3 (Cryo2)	Mouse	IgG2b	**IB:** 1:1000<br>**IF:** 1:100	Adipogen, AG-20B-0014-C100
anti-ASC	Mouse	IgG1 κ	**IB:** 1:1000<br>**IF:** 1:100	Santa Cruz Biotech, sc271054
anti-Caspase-1 (D7F10)	Rabbit	IgG	**IB:** 1:1000	Cell Signaling Technology, 3866S
anti-IL-1β	Mouse	IgG1	**IB:** 1:1000	R&D Systems, MAB201
anti-GSDMD (E951X)	Rabbit	IgG	**IB:** 1:1000	Cell Signaling Technology Cat. 39754
anti-TGN38	Rabbit	IgG	**IF:** 1:100	Novus Biological, NBP1-03495SS
anti-RCAS1 (D2B6N)	Rabbit	IgG	**IF:** 1:100	Cell signaling, 12290S
anti-γ-Tubulin	Mouse	IgG1	**IF:** 1:100	Sigma Aldrich, T5326
anti-Pericentrin	Rabbit	IgG	**IF:** 1:500	Abcam, ab4448
anti-β-actin	Mouse	IgG1	**IB:** 1:2000	Sigma Aldrich, A5441-100UL
Peroxidase-conjugated anti-Rabbit IgG (H+L)	Goat	IgG	**IB:** 1:5000 or 1:10000	Vector laboratories Inc., PI-1000
HRP-conjugated anti-Mouse IgG (H+L)	Goat	IgG	**IB:** 1:5000 or 1:10000	Promega, W402B
Alexa Fluor™ 647 anti-Rabbit IgG (H+L)	Goat	IgG	**IF:** 1:500	Invitrogen, A-21245
Alexa Fluor™ 488 anti-Rabbit IgG (H+L)	Goat	IgG	**IF:** 1:500	Invitrogen, A-11008
Alexa Fluor™ 647 anti-Mouse IgG (H+L)	Chicken	IgG	**IF:** 1:500	Invitrogen, A-21463

Supplementary references

1. Chen, J. & Chen, Z. J. PtdIns4P on dispersed trans-Golgi network mediates NLRP3 inflammasome activation. *Nature* **564**, 71–76 (2018).
2. Magupalli, V. G. *et al.* HDAC6 mediates an aggresome-like mechanism for NLRP3 and pyrin inflammasome activation. *Science (1979).* **369**, (2020).
3. Andreeva, L. *et al.* NLRP3 cages revealed by full-length mouse NLRP3 structure control pathway activation. *Cell* **184**, 6299-6312.e22 (2021).
4. Hafner-Bratkovič, I. *et al.* NLRP3 lacking the leucine-rich repeat domain can be fully activated via the canonical inflammasome pathway. *Nat. Commun.* **9**, 5182 (2018).
5. Glück, I. M. *et al.* Nanoscale organization of the endogenous ASC speck. *iScience* **26**, (2023).
6. Liu, Y. *et al.* Cryo-electron tomography of NLRP3-activated ASC complexes reveals organelle co-localization. *Nat. Commun.* **14**, (2023).
7. Bai, S., Martin-Sanchez, F., Brough, D. & Lopez-Castejon, G. Pyroptosis leads to loss of centrosomal integrity in macrophages. *Cell Death Discov.* **10**, (2024).
8. Yang, C. S. *et al.* Small heterodimer partner interacts with NLRP3 and negatively regulates activation of the NLRP3 inflammasome. *Nat. Commun.* **6**, (2015).
9. Elliott, E. I. *et al.* Cutting Edge: Mitochondrial Assembly of the NLRP3 Inflammasome Complex Is Initiated at Priming. *J. Immunol.* **200**, 3047–3052 (2018).
10. Subramanian, N., Natarajan, K., Clatworthy, M. R., Wang, Z. & Germain, R. N. The adaptor MAVS promotes NLRP3 mitochondrial localization and inflammasome activation. *Cell* https://doi.org/10.1016/j.cell.2013.02.054 (2013) doi:10.1016/j.cell.2013.02.054.
11. Kim, J. S. *et al.* Toxoplasma gondii GRA9 Regulates the Activation of NLRP3 Inflammasome to Exert Anti-Septic Effects in Mice. *Int. J. Mol. Sci.* **21**, 1–16 (2020).
12. Misawa, T. *et al.* Microtubule-driven spatial arrangement of mitochondria promotes activation of the NLRP3 inflammasome. *Nat. Immunol.* https://doi.org/10.1038/ni.2550 (2013) doi:10.1038/ni.2550.
13. Baik, S. H. *et al.* Hexokinase dissociation from mitochondria promotes oligomerization of VDAC that facilitates NLRP3 inflammasome assembly and activation. *Sci. Immunol.* **8**, (2023).
14. Zhang, Z. *et al.* Protein kinase D at the Golgi controls NLRP3 inflammasome activation. *Journal of Experimental Medicine* **214**, 2671–2693 (2017).
15. Zhou, R., Yazdi, A. S., Menu, P. & Tschopp, J. A role for mitochondria in NLRP3 inflammasome activation. *Nature* https://doi.org/10.1038/nature09663 (2011) doi:10.1038/nature09663.
16. Wang, Y. *et al.* Cellular localization of NLRP3 inflammasome. *Protein Cell* **4**, 425–431 (2013).
17. Li, X. *et al.* MARK4 regulates NLRP3 positioning and inflammasome activation through a microtubule-dependent mechanism. *Nat. Commun.* https://doi.org/10.1038/ncomms15986 (2017) doi:10.1038/ncomms15986.
18. Arumugam, S. *et al.* GSK3β mediates the spatiotemporal dynamics of NLRP3 inflammasome activation. *Cell Death Differ.* **29**, 2060–2069 (2022).
19. Zhang, Z. *et al.* Distinct changes in endosomal composition promote NLRP3 inflammasome activation. *Nat. Immunol.* **24**, 30–41 (2023).
20. Lee, B. *et al.* Disruptions in endocytic traffic contribute to the activation of the NLRP3 inflammasome. *Sci. Signal.* **16**, eabm7134 (2023).
