# Supplementary material for: Non-decameric NLRP3 reveals a TGN/MTOC-distal pathway of inflammasome activation
Source: Nat Commun. 2026 May 30;17:4866. doi: 10.1038/s41467-026-72627-x (PMC13226650; doi:10.1038/s41467-026-72627-x)
Supplement: Supplementary file 2 — Description of Additional Supplementary Files [file 41467_2026_72627_MOESM2_ESM.pdf]

## Description of Additional Supplementary Files

**Supplementary Movie 1.** Live cell imaging of LPS-primed THP-1 *NLRP3* KO cell line reconstituted with WT NLRP3 forming an NLRP3 MTOC-dependent speck upon nigericin stimulation. NLRP3 (green) and microtubules (magenta).

**Supplementary Movie 2.** Live cell imaging of LPS-primed THP-1 *NLRP3* KO cell line reconstituted with Δexon3 NLRP3 forming an NLRP3 MTOC-independent speck upon nigericin stimulation. NLRP3 (green) and microtubules (magenta).

**Supplementary Movie 3.** As in movie 1 but showing the formation of an WT NLRP3-MTOC independent speck.

**Supplementary Movie 4.** As in movie 1 but showing the formation of an WT NLRP3-MTOC independent speck in the presence of nocodazole.
