# Supplementary material for: Non-decameric NLRP3 reveals a TGN/MTOC-distal pathway of inflammasome activation
Source: Nat Commun. 2026 May 30;17:4866. doi: 10.1038/s41467-026-72627-x (PMC13226650; doi:10.1038/s41467-026-72627-x)
Supplement: Supplementary file 7 — Reporting Summary [file 41467_2026_72627_MOESM7_ESM.pdf]

Corresponding author(s): Alexander N. R. Weber and Ana Tapia-Abellán

Last updated by author(s): Apr 1, 2026

Reporting Summary

Nature Portfolio wishes to improve the reproducibility of the work that we publish. This form provides structure for consistency and transparency in reporting. For further information on Nature Portfolio policies, see our Editorial Policies and the Editorial Policy Checklist.

Statistics

For all statistical analyses, confirm that the following items are present in the figure legend, table legend, main text, or Methods section.

n/a	Confirmed	
☐	☒	The exact sample size (*n*) for each experimental group/condition, given as a discrete number and unit of measurement
☐	☒	A statement on whether measurements were taken from distinct samples or whether the same sample was measured repeatedly
☐	☒	The statistical test(s) used AND whether they are one- or two-sided<br>*Only common tests should be described solely by name; describe more complex techniques in the Methods section.*
☒	☐	A description of all covariates tested
☐	☒	A description of any assumptions or corrections, such as tests of normality and adjustment for multiple comparisons
☐	☒	A full description of the statistical parameters including central tendency (e.g. means) or other basic estimates (e.g. regression coefficient) AND variation (e.g. standard deviation) or associated estimates of uncertainty (e.g. confidence intervals)
☐	☒	For null hypothesis testing, the test statistic (e.g. *F*, *t*, *r*) with confidence intervals, effect sizes, degrees of freedom and *P* value noted<br>*Give P values as exact values whenever suitable.*
☒	☐	For Bayesian analysis, information on the choice of priors and Markov chain Monte Carlo settings
☒	☐	For hierarchical and complex designs, identification of the appropriate level for tests and full reporting of outcomes
☐	☒	Estimates of effect sizes (e.g. Cohen's *d*, Pearson's *r*), indicating how they were calculated

Our web collection on statistics for biologists contains articles on many of the points above.

Software and code

Policy information about availability of computer code

Data collection	For microscopy the ZEN Blue software from Zeiss LSM800 confocal (Zeiss, Germany)was used. ELISAs and cell death assays were measured in Gene5 & Gene 5 Secure (Synergy Neo2, Biotek Instruments, USA). Negative stain images from a Jeol JEM-2200FS transmission electron microscope. Gel filtration assays inin the ÄKTA Pure system (Cytiva). Potential targets sites for efficient exon 3 skipping were identified using the Human Splicing Finder server (GENOMNIS SAS Company, France). QuantStudio Real-Time-PCR software version 1.3 (Thermo Fisher Scientific) for qPCR. Western blots images were acquired using Image Studio v5.2 from LI-COR (Odyssey).Electroporation was performed in the Lonza Nucleofector 2b. The search of NLRP3 lacking exon3 inin a human setting was done in 186 human RNA-Seq samples from seven NCBI BioProjects (accession IDs: PRJNA378936; PRJNA674655; PRJNA722048; PRJNA789541; PRJNA901389; PRJNA941263; PRJNA978574).
Data analysis	Preparation of graphs, experimental data analysis and statistics were done using Prism (GraphPad Inc., v8). Microscopy data were analysed using Fiji/ImageJ (US National Institutes ofof Health, Bethesda, MDMD USA). Multi-angle light scattering (MALS) data analysis was done using the ASTRA 8 software (WYATT technologies). Western blots images were analysed using Image Studio v5.2 from LI-COR (Odyssey). The predicted mRNA structure was analysed using the mFoldWeb Server The analysis of NLRP3 lacking exon3 from 186 human RNA-Seq samples was done using NCBI SRA nucleotide BLAST.

For manuscripts utilizing custom algorithms or software that are central to the research but not yet described in published literature, software must be made available to editors and reviewers. We strongly encourage code deposition in a community repository (e.g. GitHub). See the Nature Portfolio guidelines for submitting code & software for further information.

Data

Policy information about availability of data

All manuscripts must include a data availability statement. This statement should provide the following information, where applicable:

- Accession codes, unique identifiers, or web links for publicly available datasets
- A description of any restrictions on data availability
- For clinical datasets or third party data, please ensure that the statement adheres to our policy

All materials and data generated during this study are included in this Article and its Supplemental Information files or available from the authors upon request, as are unique reagents used in this Article. The raw numbers for charts and graphs and the uncropped western blots are available in the Source Data file.

Research involving human participants, their data, or biological material

Policy information about studies with human participants or human data. See also policy information about sex, gender (identity/presentation), and sexual orientation and race, ethnicity and racism.

Reporting on sex and gender	Sex and gender were not taking into account for the healthy donors used in this study.
Reporting on race, ethnicity, or other socially relevant groupings	Race and ethnicity analysis were not included in the informed consent and was not collected.
Population characteristics	Healthy blood donors were used in this study.
Recruitment	Healthy donors were recruited at the Institute of Immunology (Tübingen) and Hospital General Universitario Santa Lucía (Cartagena, Spain).
Ethics oversight	Approval of the Ethical Committee of the Medical Faculty Tübingen (Tübingen, Germany) and Hospital General Universitario Santa Lucia (Cartagena, Spain).

Note that full information on the approval of the study protocol must also be provided in the manuscript.

Field-specific reporting

Please select the one below that is the best fit for your research. If you are not sure, read the appropriate sections before making your selection.

☒ Life sciences ☐ Behavioural & social sciences ☐ Ecological, evolutionary & environmental sciences

For a reference copy of the document with all sections, see nature.com/documents/nr-reporting-summary-flat.pdf

Life sciences study design

All studies must disclose on these points even when the disclosure is negative.

Sample size	No sample size calculations were performed.
Data exclusions	Outliers were assessed and removed in figure Fig. 6A and supplementary Fig. 6A using robust regression and outlier removal (ROUT) using a false discovery rate (FDR) value of 0.01.
Replication	Experiments were repeated at least three times and/or with sufficient cells per group to demonstrate statistical significance using identical protocols and settings as indicated in each figure legend. All replication experiments give a similar result as the presented representative in main figures. Negative stain and SEC-MALS results come from NLRP3 proteins purified once.
Randomization	Randomization is not considered relevant for in vitro assays.
Blinding	Blinding is not considered relevant for the in vitro assays presented in this study, at the risk of bias is already minimized by the controlled environment where the experiments were performed and the use of negative and positive controls. For microscopy experiments in fixed or living cells the images were randomly acquired with the same settings. We declare that the experiments were conducted with the high level of rigor and transparency following reported methods.

Reporting for specific materials, systems and methods

We require information from authors about some types of materials, experimental systems and methods used in many studies. Here, indicate whether each material, system or method listed is relevant to your study. If you are not sure if a list item applies to your research, read the appropriate section before selecting a response.

Materials & experimental systems

n/a	Involved in the study	
☐	☒	Antibodies
☐	☒	Eukaryotic cell lines
☒	☐	Palaeontology and archaeology
☒	☐	Animals and other organisms
☒	☐	Clinical data
☒	☐	Dual use research of concern
☒	☐	Plants

Methods

n/a	Involved in the study	
☒	☐	ChIP-seq
☐	☒	Flow cytometry
☒	☐	MRI-based neuroimaging

Antibodies

Antibodies used

Antibody name: Rabbit anti-NLRP3 mAb
Supplier name: Cell Signaling Technology
Catalogue number: 15101
Clone name: D4D8T

Antibody name: Mouse anti-NLRP3 mAb
Supplier name: Adipogen
Catalogue number: AG-20B-0014-C100
Clone name: Cryo2

Antibody name: Mouse anti-ASC mAb
Supplier name: Santa Cruz Biotech
Catalogue number: sc271054
Clone name: F-9

Antibody name: Rabbit anti-caspase-1 mAb
Supplier name: Cell Signaling Technology
Catalogue number: 3866
Clone name: D7F10

Antibody name: Mouse anti-IL-1beta mAb
Supplier name: R&D Systems
Catalogue number: MAB201
Clone name: 8516

Antibody name: Rabbit anti-GSDMD mAb
Supplier name: Cell Signaling Technology
Catalogue number: 39754
Clone name: E9S1X

Antibody name: Rabbit anti-TGN38 pAb
Supplier name: Novus Biological
Catalogue number: NBP1-03495SS

Antibody name: Rabbit anti-RCAS1 mAb
Supplier name: Cell signaling Technology
Catalogue number: 12290S
Clone name: D2B6N

Antibody name: Mouse anti-Gamma-Tubulin mAb
Supplier name: Sigma Aldrich
Catalogue number: T5326
Clone name: GTU-88

Antibody name: Mouse anti-beta-Actin mAb
Supplier name: Sigma Aldrich
Catalogue number: A5441
Clone name: AC-15

Antibody name: Rabbit anti-Pericentrin pAb
Supplier name: Abcam
Catalogue number: AB4448

Antibody name: Goat Peroxidase-conjugated Anti-Rabbit IgG Antibody (H+L)
Supplier name: Vector laboratories Inc
Catalogue number: PI-1000

Antibody name: Anti-Mouse IgG (H+L) HRP-conjugated
Supplier name: Promega
Catalogue number: W402B

Antibody name: Goat Alexa Fluor™ 647 anti-Rabbit IgG (H+L)
Supplier name: Invitrogen
Catalogue number: A-21245

Antibody name: Goat Alexa Fluor™ 488 anti-Rabbit IgG (H+L)
Supplier name: Invitrogen
Catalogue number: A-11008

Antibody name: Chicken Alexa Fluor™ 647 anti-Mouse IgG (H+L)
Supplier name: Invitrogen
Catalogue number: A-21463

Validation

All commercial antibodies were from commercial suppliers and verified according to manufacturer´s specification on their corresponding websites or by other authors.

Antibody name: Rabbit anti-NLRP3 mAb (D4D8T)
Specificity: human, mouse. This antibody recognizes endogenous levels of total NLRP3 protein in residues surrounding Ala306 of mouse NLRP3 protein.
Application: WB 1:1000 dilution

Antibody name: Mouse anti-NLRP3 mAb (Cryo2)
Specificity: human, mouse. The antibody recognizes an epitope located in the PYD domain (aa 1-93) of mouse NLRP3
Application: IF (Sci Rep. 6, 24667, 2016) dilution 1:200. For native NLRP3 detection on lipid strips (Nature. 604, 184–189, 2022) dilution 1:1000

Antibody name: Mouse anti-ASC mAb
Specificity: mouse, rat and human. His antibody is raised against amino acids 1-120 mapping at the N-terminus of ASC of human origin
Application: WB dilution 1:1000, IF dilution 1:200

Antibody name: Rabbit anti-caspase-1 mAb
Specificity: human. The antibody detects endogenous levels of full-length human Caspase-1. The activated p20 subunit was detected by over-expression.
Application: WB dilution 1:1000

Antibody name: Mouse anti-IL-1Beta mAb
Specificity: human.
Application: WB dilution 1:1000

Antibody name: Rabbit anti-GSDMD mAb
Specificity: mouse, rat and human. The antibody recognizes endogenous levels of total Gasdermin D protein. This antibody recognizes the 30 kDa amino terminal fragment produced during pyroptosis by caspase-1.
Application: WB dilution 1:1000

Antibody name: Rabbit anti-TGN38 pAb
Specificity: mouse, rat, primate and human
Application: IF 1:200

Antibody name: Rabbit anti-RCAS1 mAb
Specificity: mouse, rat and human. The antibody recognizes endogenous levels of total RCAS1 protein in the residues surrounding Gly 147 of human RCAS1 protein.
Application: IF 1:200

Antibody name: Mouse anti-gamma-Tubulin mAb
Specificity: Human, Mouse. The exact immunogen used to generate this antibody is proprietary information.
region of gamma-tubulin.
Application: IF 1:200

Antibody name: Rabbit anti-pericentrin pAb

Specificity: Human, mouse
Application: IF 1:500

Antibody name: Mouse anti-Beta-actin mAb
Specificity: sheep, carp, feline, chicken, rat, mouse, Hirudo medicinalis, rabbit, canine, pig, human, bovine, guinea pig. The antibody recognises an epitope in the N-terminal region of the Beta-isoform of actin.
Application: WB 1:2000

Eukaryotic cell lines

Policy information about cell lines and Sex and Gender in Research

Cell line source(s)	THP-1 cell line was gift of Thomas Zillinger; University of Bonn. THP-1 Null 2 and THP-1 Null 2 NLRP3 KO cell lines were a gift of Gloria López Castejón University of Manchester and come from InvivoGen (Cat#thp-nullz and Cat#thp-konlrp3z, respectively). THP-1 Null 2 NLRP3 KO reconstituted with NLRP3 WT-mNG or NLRP3 lacking exon3-mNG were generated and validated in this paper. HeLa was gift of Hans-Georg Rammensee, University of Tübingen. HeLa NLRP3 WT-mNG, HeLa NLRP3 lacking exon3-mNG and HeLa NLRP3 AMAA-mNG were generated and validated in this paper. Sf9 cell line comes from Thermo Fisher Scientific Cat#12659017
Authentication	None of the cell lines used were authenticated but cellular identity was regularly checked by morphology and regular cell behavior
Mycoplasma contamination	All cell lines used were tested prior used and on a regular basis and showed no mycoplasma contamination
Commonly misidentified lines (See ICLAC register)	none

Plants

Seed stocks	*Report on the source of all seed stocks or other plant material used. If applicable, state the seed stock centre and catalogue number. If plant specimens were collected from the field, describe the collection location, date and sampling procedures.*
Novel plant genotypes	*Describe the methods by which all novel plant genotypes were produced. This includes those generated by transgenic approaches, gene editing, chemical/radiation-based mutagenesis and hybridization. For transgenic lines, describe the transformation method, the number of independent lines analyzed and the generation upon which experiments were performed. For gene-edited lines, describe the editor used, the endogenous sequence targeted for editing, the targeting guide RNA sequence (if applicable) and how the editor was applied.*
Authentication	*Describe any authentication procedures for each seed stock used or novel genotype generated. Describe any experiments used to assess the effect of a mutation and, where applicable, how potential secondary effects (e.g. second site T-DNA insertions, mosiacism, off-target gene editing) were examined.*

Flow Cytometry

Plots

Confirm that:

- [x] The axis labels state the marker and fluorochrome used (e.g. CD4-FITC).
- [x] The axis scales are clearly visible. Include numbers along axes only for bottom left plot of group (a 'group' is an analysis of identical markers).
- [x] All plots are contour plots with outliers or pseudocolor plots.
- [x] A numerical value for number of cells or percentage (with statistics) is provided.

Methodology

Sample preparation	After PMN isolation and stimulation, the purity and activation status of neutrophils wsa determined by flow cytometry. 200 uL of the cell suspension was transferred into 96 well plate (U-shape) ans spun down for 5 min at 450 x g 4 ºC. FcR block was performed using pooled human serum diluted 1:10 in FACS buffer (PBS, 1mM EDTA, 2 % heat inactivated FBS) for 15 min at 4 ºC. After washing, the samples were stained for approximately 20-30 min at 4 ºC in the dark. Thereafter, fixation buffer (4 % PFA in PBS) was added to the cells for 10 min at RT in the dark. After an additional washind step, the cell pellet were resuspended in 150 uL FACS buffer. For sorting experiments, THP-1 NLRP3 KO cells and THP-1 NLRP3 KO cells stably expressing NLRP3 WT-mNG or Δexon3-mNG were prepared by centrifuging one T75 cm2 flask per cell line, resuspending them in phenol red-free RPMI supplemented with 10 % FBS at 1 × 10^7 cells per 100 µL, and passing the suspension through a cell strainer immediately before sorting.
Instrument	PMN measurements were performed on a FACS Canto II from BD Bioscience THP-1 NLRP3 KO cells stably expressing NLRP3 WT-mNG or Δexon3-mNG sorting were performed on a BD FACSAria III using FACSDiva v9.0.1
Software	BD Bioscence, Diva Software. Analysis was performed using FlowJo V10 analysis software

Cell population abundance

more than 95% after isolation for PMNs and THP-1

Gating strategy

PMNs were gated by FSC-A/SSC-A and doublets were excluded. Live cells were gated using thre following markers. In CD15/CD66b double positive cells and CD14 low/negative were considered PMNs. CD62L was used as an early activation marker. For sorting experiments, THP-1 cells stably expressing NLRP3-WT or NLRP3 lacking exon 3, both tagged with mNeonGreen (mNG) were gated based on mNG fluorescence, following singlet discrimination. mNG-positive cells were selected and subsequently sorted into low, medium and high expression populations according to fluorescence intensity. An unstained THP-1 control was used to define background fluorescence and set the gates

☒ Tick this box to confirm that a figure exemplifying the gating strategy is provided in the Supplementary Information.
